# Aquagenic urticaria: Water, friend, or foe?

**DOI:** 10.1002/ccr3.2880

**Published:** 2020-09-24

**Authors:** Arturo Robles‐Tenorio, Victor Manuel Tarango‐Martinez, Georgina Sierra‐Silva

**Affiliations:** ^1^ CUCS Universidad de Guadalajara Guadalajara México; ^2^ Instituto Dermatologico de Jalisco “Dr. José Barba Rubio” (IDJ) Zapopan México

**Keywords:** allergy, aquagenic, urticaria, water

## Abstract

There are <100 reported cases of aquagenic urticaria. Although most are sporadic, several diseases have been associated. Diagnosis is based on provocation tests. Second‐generation antihistamines are the first‐line treatment.

## Graphical abstract

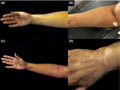

## 1 INTRODUCTION

Water, one of the fundamental symbols of life, can become a nuisance for some individuals. Aquagenic urticaria (AU) is an extraordinary type of physical or chronic inducible urticaria (CI‐U) elicited by water exposure. With around 100 cases been described in the literature, the disease has received little to no attention by the scientific community. Presently, pathogenesis remains unknown. The diagnosis is supported by clinical history and confirmed by provocation tests, which include the discrimination from other CI‐Us. Since trigger avoidance is nearly impossible, treatment is based on symptomatic control.

Here, we present the first case of sporadic AU in Mexico, as well as an updated revision of the relevant literature. This work contributes to the scant reports documented in the Latin‐American population.^1^, ^2^ Our patient showed an adequate therapeutic response to a second‐generation antihistamine and a skin barrier–repairing cream. Treatment efficacy was assessed by applying two validated questionnaires aimed at measuring disease‐related symptom severity and quality of life (QoL).

## 2 CASE REPORT

An otherwise healthy 53‐year‐old Mexican female patient presented with a 40‐year history of generalized pruritus and wheal formation during bathing and dishwashing, which lasted for 30‐60 minutes after ceasing water contact. The patient had been previously self‐treated with unspecified cleansers, soaps, and commercial creams, noting variable but insufficient relief. Symptoms did not occur during physical activity, emotionally stressful situations, or exposure to temperature changes. Angioedema and dyspnea had never been present during active disease or under any other circumstance. The patient denied previous history of asthma, atopy, or allergies. No family members were affected.

**Figure 1:**
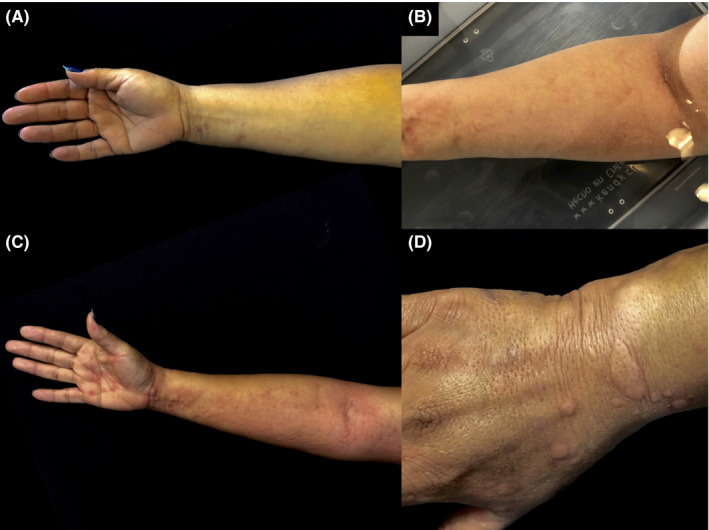
A, Initial clinical appearance. B, Right arm water immersion (from the hand up to the elbow fossa) triggered the development of pruritus, erythema, and wheals at room temperature. C, A clear urticarial reaction was observed on the ventral and D, dorsal aspect of the right arm, exclusively affecting water‐exposed areas

On physical examination, skin appeared clear and dermographism was negative. In order to induce the appearance of lesions, water‐provocation tests were performed according to current recommendations.^3^, ^4^, ^5^, ^6^ Wet compresses were applied for 20 minutes, yielding a negative result. Next, in‐office water immersion of the right arm was performed. After 5 minutes, multiple wheals, intense erythema, and pruritus developed over the wet area (Figure 1). Cold and heat urticaria were excluded by exposing the patient to an icepack and a heating pad, as per suggested by urticaria guidelines.^3^, ^4^, ^6^ Testing for other types of CI‐Us was omitted based on the history of present illness and the positive water‐challenge test. Accordingly, the diagnosis of sporadic AU was established.

Symptomatic treatment was initiated with cetirizine 10 mg daily and frequent use of a ceramide‐containing moisturizing cream. After 1 month of treatment, cetirizine was increased to 10 mg twice daily to achieve symptom control. At the 4‐month follow‐up visit, the patient referred marked improvement. This was evaluated by applying the Chronic Urticaria Quality of Life Questionnaire (CU‐Q_2_oL) and the Dermatology Life Quality Index (DLQI), observing a decrease from 28 to 4 and 14 to 5 points from the initial visit, respectively. No adverse effects were noted.

## 3 DISCUSSION

AU was first described in 1964^7^ and belongs to the spectrum of aquagenic disorders.^5^ Due to its rarity, prevalence has not been defined. Only a couple of cases have been documented in Latin America,^1^, ^2^ but none have been previously described in Mexico. Disease under‐recognition, rather than a lack of affected subjects, may partly explain the scarcity of reported cases.

From the available literature, a female predominance has been shown over males.^5^ The condition typically develops during puberty and shows a chronic behavior.^8^ For most individuals, disease occurs sporadically and without any accompanying diseases. All of the above is consistent with our case presentation. Nonetheless, isolated reports have documented familial occurrence^9^, ^10^, ^11^ and concomitance with Bernard‐Soulier syndrome,^12^ polymorphic light eruption,^13^ familial lactose intolerance,^11^ and papillary carcinoma of the thyroid.^14^ Due to the paucity of these observations, it remains unknown whether these conditions share a common background or whether there are various predisposing factors (genetic and/or environmental). Therefore, performing a thorough investigation of personal and family medical history is strongly encouraged.

Three hypotheses have been formulated aimed at explaining the pathogenesis. The first proposed that water reaction with sebum or sebaceous glands forms a toxic substance that results in mast cell degranulation.^15^ Then, it was thought that undefined water‐soluble epidermal antigens diffuse across the dermis causing histamine release.^16^ Lastly, histamine‐independent mechanisms were suggested after observing unchanged serum histamine levels in a patient with AU.^17^ Regarding water properties, salt‐dependent AU (SDAU) has been described in <10 cases,^18^, ^19^ whereas temperature and pH have not shown an influence.^15^

The diagnosis of AU is prompted by medical history and confirmed by water‐challenge tests.^3^, ^5^ Accompanying syncope and dyspnoea^14^, ^20^ are uncommon. Manifestations cease shortly after discontinuing water contact. Dermographism, angioedema, or wheezing must be ruled out on physical examination. The differential diagnoses include other forms of CI‐Us (heat, cold, and cholinergic) and aquagenic pruritus. The EAACI/GA(2) LEN/EDF/WAO guideline establishes that room‐temperature wet clothes must be applied for 20 minutes,^3^ yet water immersion can be performed if the former is negative.^5^ Pruritus, erythema, and wheals indicate a positive reaction and confirm the diagnosis. For patients referring symptoms only after sea water exposure (SDAU), a 3.5% NaCl solution must be utilized.^18^, ^19^ Additional routine laboratory examinations and/or skin biopsies are not warranted. If performed, a biopsy should immediately follow a positive provocation test. Histopathological features are nonspecific and demonstrate a mixed interstitial dermal infiltrate and mild dermal edema.^5^

In our patient, medical history clearly suggested an aquagenic disorder. The appearance of wheals after the water‐challenge test ruled out the possibility of aquagenic pruritus and other forms of CI‐Us. Despite this, we excluded the effects of temperature in urticarial reaction development. The fact that a positive reaction was only evident after water immersion suggests that epidermal barrier disruption may play an important role in disease pathogenesis. This observation contributes to the previously generated hypotheses, with relevant implications in skin barrier restoration for therapeutic purposes.

Since trigger avoidance is not feasible, management is focused on symptom management. Second‐generation antihistamines are the first‐line of therapy, with doses that can be increased up to fourfold.^3^ Cetirizine is a selective H_1_ receptor antagonist known to reduce wheal formation and modulate the inflammatory cascade through several pathways.^21^ Peak response is reached 4‐8 hours after ingestion. The half‐life of a single 10 mg dose ranges between 6.7 and 8.6 hours. Since water exposure is constant throughout the day, it is reasonable that our patient required a 10 mg twice daily dosage to achieve an adequate therapeutic response.

Topical barrier moisturizers can be used adjunctively. From these, ceramide‐containing creams constitute acceptable alternatives. Ceramides are important lipids of the stratum corneum that participate in skin barrier maintenance, cell adhesion, proliferation, epidermal differentiation, and apoptosis.^22^, ^23^ Recently, the efficacy of a ceramide moisturizing cream on skin hydration and barrier function was objectively measured through corneometry, transepidermal water loss, and pH assessment in senile xerosis patients.^24^ Interestingly, increased skin hydration, reduced transepidermal water loss, and pH decline were observed for up to 24 hours after a single application. Improvement was greater at a 28‐day time point, following a twice‐daily administration. More specifically, a protective oil‐in‐water emulsion has reportedly shown suppressive effects on rash and pruritus in a patient with AU, when used in combination with 10 mg daily of cetirizine.^25^ Thus, we believe that skin barrier restoration may also constitute an important therapeutic principle for AU. According to this, well‐conducted skin barrier research might prove useful for a deeper understanding of disease mechanisms.

Other treatment alternatives include first‐generation antihistamines, antileukotrienes, phototherapy, and omalizumab.^26^ Case‐specific evidence exists for stanozolol in a patient with AU, angioedema, and HIV infection.^27^

Prognosis after treatment initiation is variable. Hence, AU management remains a therapeutic challenge. Current guidelines^3^ suggest applying the CU‐Q_2_oL^28^ instrument for monitoring disease activity and QoL in patients with chronic urticaria. In our case, we used the validated Spanish version.^29^ The questionnaire includes 23 items that evaluate symptom severity and life quality. In addition, we confirmed this by using the DLQI^30^ questionnaire. To our knowledge, previously reported AU cases have not included objective measures to assess therapeutic efficacy. Since management recommendations remain anecdotal, quantitative evaluation of treatment responses should be a vital consideration for future reports.

This case contributes to the scant publications of worldwide AU prevalence and aims to illuminate our current understanding of this extraordinary reaction. Despite most cases being sporadic, we have highlighted associated diseases and the diagnostic approach to urge recognition of this entity. Lastly, we believe that skin barrier research may reveal unexplored pathogenic mechanisms, which could possibly yield more targeted treatment suggestions.

## CONFLICT OF INTEREST

The authors declare that there is no conflict of interest regarding the publication of this article.

## AUTHOR CONTRIBUTIONS

All authors: involved in the writing, revisions, and final review of the manuscript.
